# Visual Detection of Dengue-1 RNA Using Gold Nanoparticle-Based Lateral Flow Biosensor

**DOI:** 10.3390/diagnostics9030074

**Published:** 2019-07-11

**Authors:** Flora M. Yrad, Josephine M. Castañares, Evangelyn C. Alocilja

**Affiliations:** 1 Nano-Biosensors Lab, Department of Biosystems and Agricultural Engineering, Michigan State University, East Lansing, MI 48824, USA; 2 Department of Chemistry, University of San Carlos, Talamban, Cebu 6000, Philippines; 3 Department of Chemistry, Silliman University, Dumaguete 6200, Philippines

**Keywords:** dengue-1, dextrin-capped AuNP, lateral flow biosensor, NASBA, sandwich-type hybridization

## Abstract

Dengue is a rapidly spreading mosquito-borne viral disease. Early diagnosis is important for clinical screening, medical management, and disease surveillance. The objective of this study was to develop a colorimetric lateral flow biosensor (LFB) for the visual detection of dengue-1 RNA using dextrin-capped gold nanoparticle (AuNP) as label. The detection was based on nucleic acid sandwich-type hybridization among AuNP-labeled DNA reporter probe, dengue-1 target RNA, and dengue-1 specific DNA capture probe immobilized on the nitrocellulose membrane. Positive test generated a red test line on the LFB strip, which enabled visual detection. The optimized biosensor has a cut-off value of 0.01 µM using synthetic dengue-1 target. Proof-of-concept application of the biosensor detected dengue-1 virus in pooled human sera with a cut-off value of 1.2 × 10^4^ pfu/mL. The extracted viral RNA, when coupled with nucleic acid sequence-based amplification (NASBA), was detected on the LFB in 20 min. This study first demonstrates the applicability of dextrin-capped AuNP as label for lateral flow assay. The biosensor being developed provides a promising diagnostic platform for early detection of dengue infection in high-risk resource-limited areas.

## 1. Introduction

Dengue is a mosquito-borne disease that affects more than 100 countries in the tropical and sub-tropical regions [1]. An estimated 50–100 million people are infected annually with 250,000–500,000 severe cases and 24,000 deaths [2]. Dengue disease is caused by the single-stranded, positive-sense RNA dengue virus (DENV) that occurs in four distinct serotypes (DENV-1, DENV-2, DENV-3, and DENV-4) [3,4]. Primary infection with any of the serotypes can cause dengue fever (DF) that may progress to life-threatening dengue hemorrhagic fever (DHF) and dengue shock syndrome (DSS). Secondary infection with other heterologous type increases the risk of developing DHF and DSS [5,6].

Diagnosis of dengue disease based solely on clinical symptoms is difficult and unreliable because initial symptoms of infection are similar to influenza, measles, yellow fever, and other viral infections [7,8]. Laboratory diagnosis for dengue disease is therefore important for clinical screening, better medical management, and disease surveillance. Current methods used to confirm dengue infection include virus isolation, serological, and molecular techniques [9]. Reverse transcriptase polymerase chain reaction (RT-PCR) remains the gold standard for sensitive molecular and serotyping of dengue virus, but this technique is expensive [10] and rare in endemic areas. Reliance on PCR and fluorescent detection contribute to the cost and complexity of nucleic acid diagnostics [11]. There is a need for a simple, rapid, and affordable detection method that will provide confirmatory result especially in high-risk, resource-limited areas.

Lateral flow biosensor (LFB) is a paper-based testing device that performs rapid assay in one step and simple format. It is a pre-fabricated strip containing dry reagents that are activated when fluid samples are applied. It is designed for single use and suitable for applications where positive/negative signal is sufficient. Recently, LFB has attracted major interests because of its promising tool in point-of-care (POC) diagnostics that meet the ASSURED criteria (affordable, sensitive, specific, user-friendly, rapid/robust, equipment–free, and deliverable to end users) [12]. Many reported LFBs use AuNP as label because of its unique properties [13,14]. Its vivid red color makes detection by the naked eye easy and the sensitivity of the lateral flow assay is related to the intensity of the red color on the test line [15,16].

**Scheme 1:**
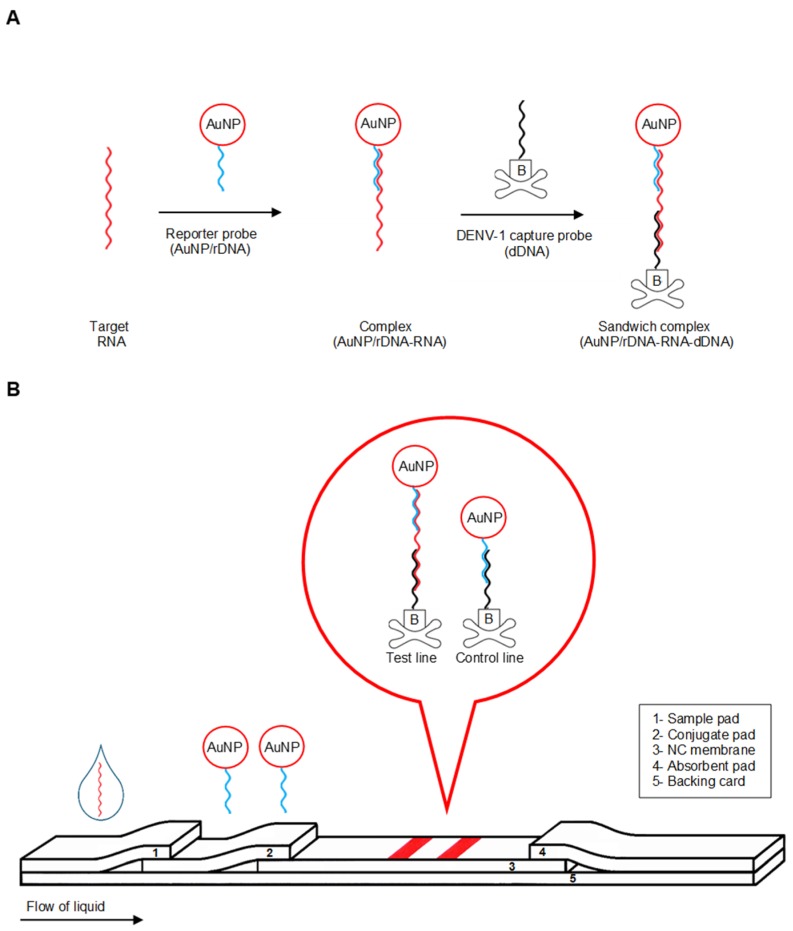
Illustration of detection mechanism. (**A**) Formation of AuNP/rDNA-RNA-dDNA sandwich complex. (**B**) Schematic of visual detection.

The objective of this study was to develop a lateral flow biosensor for visual detection of dengue-1 RNA using dextrin-capped AuNP as label. The biosensor used three DNA probes, namely AuNP-labeled reporter probe (AuNP/rDNA), capture probe specific to DENV-1 (dDNA), and control probe (cDNA), the latter two being dispensed on the nitrocellulose membrane, which formed the test and control lines, respectively. The probes were designed to detect negative-sense target RNA amplicon from nucleic acid sequence-based amplification (NASBA). An advantage of NASBA over RT-PCR is that, it is an RNA amplification that involved isothermal reactions at 41 °C without the need of thermocycling [17]. The detection was based on nucleic acid sandwich-type hybridization reactions between AuNP/rDNA, target RNA, and dDNA on the test line of the LFB strip. The detection mechanism is illustrated in Scheme 1.

## 2. Materials and Methods

### 2.1. Materials and Chemicals

Cellulose fiber pad (CFSP17300, 17 mm × 300 mm), glass fiber pad (GFCP 103000, 10 mm × 300 mm), and nitrocellulose membranes (HF 135 and HF180) adhered on backing cards (60 mm × 301 mm) were purchased from EMD Millipore (Taunton, MA, USA). Lyophilized powder of pooled normal human sera from clotted whole blood was purchased from Sigma-Aldrich and reconstituted using Type I water. Nuclease-free water was purchased from Integrated DNA Technologies, Inc. (IDT). Other chemicals were obtained commercially and of analytical grade. All aqueous solutions were prepared using Type I water (≤18.2 MΩ cm) from Direct-Q3 Water Purification System. 

### 2.2. Oligonucleotides

All oligonucleotides were purchased from IDT (Coralville, IA, USA). DENV-1 capture and reporter probes (dDNA and rDNA) for detection, and primers (P1 and P2) for amplification were previously designed [18]. The control probe (cDNA) was designed in this study as a complimentary strand to the reporter probe. The oligonucleotides for the sandwich hybridization assay were designed to detect NASBA product of the RNA target. Their sequences were provided in Table 1.

The dDNA sequence corresponded to base positions 10,550–10,574 of DENV-1 genome Singapore strain (Gen Bank accession number M87512). P1 and P2 were complementary to bases 10,632–10,653 and bases 10,497–10,516 of the DENV-1 genome, respectively. Synthetic DENV-1, -2, -3, and -4 target analytes (T-1, T-2, T-3, and T-4) corresponded to base positions 10497–10653 of Dengue-1 Singapore strain; 10501–10659 of Jamaica/N.1409 (GenBank accession number M20558); 10477–10632 of H87 isolate (GenBank accession number M93130); and 10424–10584 of dengue-4 (GenBank accession number M14931), respectively. The sizes of T-1, T-2, T-3, and T-4 were 157, 159, 156, and 160 DNA bases, respectively. DENV-1 synthetic target RNA (T-RNA) was also used. T-RNA corresponded to base positions 10491–10532, 10543–10580, and 10621–10660 of DENV-1 Singapore strain combined together and formed a single strand of 120-base length RNA oligonucleotide that incorporated complementary sequences for P1, dDNA, and P2, respectively. Synthetic Chikungunya and Zika target analytes, T-CHIKV and T-ZIKV corresponded to base positions 3962–4133 of strain TSI-GSD-218 (GenBank accession number L37661) and 9271–9373 (GenBank accession number NC_012532), respectively. T-CHIKV and T-ZIKV were 192 and 123 bases long, respectively; each of their 3’ ends contained an overhang of 20-base long sequence complimentary to the AuNP-labeled reporter probe.

### 2.3. Preparation of AuNP and AuNP/rDNA Conjugate

AuNPs with 10 nm mean core size were prepared by modifying the dextrin method [19]. Into a 125 mL Erlenmeyer flask, 39.5 mL water, 5 mL of 20 mM HAuCl_4_, 0.5 mL 10% sodium carbonate, and 5 mL of 25 g/L dextrin were added sequentially. The Erlenmeyer flask wrapped in aluminum foil was heated with stirring for one hour in an isotemp hotplate/stirrer (Fisher Scientific) at 150 °C temperature setting and then cooled down to room temperature. The morphology and size of the synthesized AuNP were evaluated using UV-vis spectrophotometry and high-resolution transmission electron microscopy. One mL of the synthesized AuNP, concentrated 2X, was functionalized with thiolated rDNA as previously described [20] using 50 µL of 100 µM rDNA and 50 µL of freshly prepared 0.2 M DTT. The AuNP/rDNA conjugate produced was purified by centrifugation at 13,000 rpm for 15 min at 4 °C using a centrifuge (Eppendorf 5424 R). The supernatant was removed and the pellet was washed with 10 mM phosphate buffer saline (PBS) twice. The washed pellet was resuspended in phosphate–bovine serum albumin (BSA) buffer (20 mM Na_3_PO_4_, 5% BSA, 0.25% Tween, and 10% sucrose) [21] either retaining the original volume or further diluted as desired. Purified AuNP/rDNA conjugate was stored at 4 °C until use.

### 2.4. Immobilization of dDNA and cDNA Probes 

The 5’-biotinylated dDNA and 3’-biotinylated cDNA were reacted with streptavidin and formed the dDNA and cDNA conjugates intended for the test and control lines, respectively. Amounts of 100 µL of 10 mM PBS and 200 µL of 1 mg/mL streptavidin were added to 100 µL of 100 µM biotinylated probe. The mixture was incubated for 1 h at room temperature with shaking and filtered using EMD Millipore Amicon^®^ Ultra-0.5 Centrifugal Filter device 3K (Taunton, MA, USA). The conjugate was washed three times with 200 µL 10 mM PBS each time in the same centrifugal filter. The purified conjugate was stored at 4 °C until use.

### 2.5. Assembly of LFB

**Figure 1:**
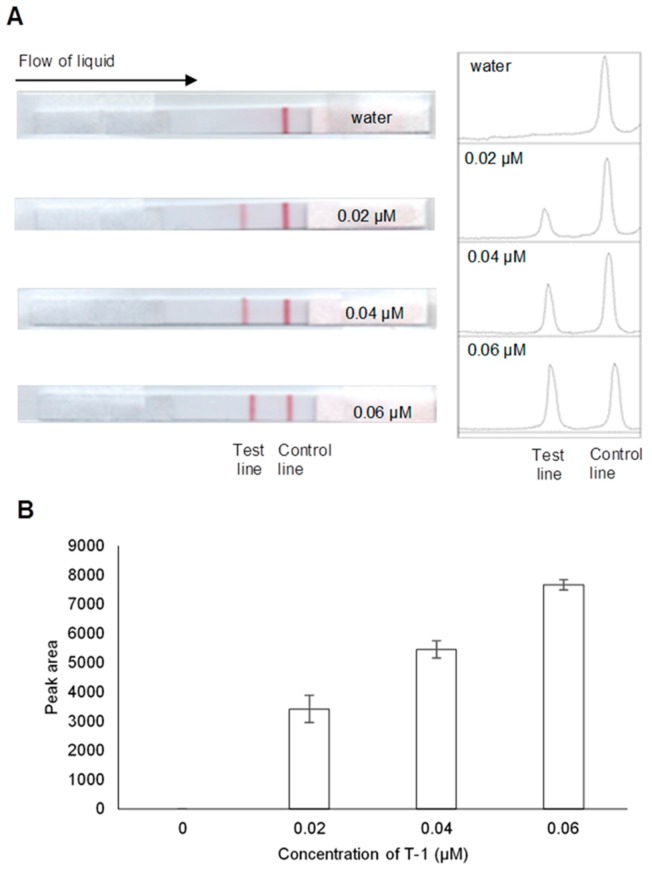
(**A**) Photo images of valid lateral flow biosensor (LFB) results at indicated concentrations of T-1 and their corresponding profile plots generated from ImageJ analysis. (**B**) Histogram representing peak areas of the test lines responding to 0 (water), 0.02, 0.04, and 0.06 µM T-1. Error bars represent standard deviation (*n* = 3).

The LFB consisted of four components constructed as shown in Figure 1B. Each fiberglass sample pad was wetted with 2 mL of Tris-HCl buffer (pH 8.0) containing 0.05 M Tris-HCl, 0.25% Triton X-100, and 0.15 M NaCl [19]. After 30 min of soaking, the pad was dried in the vacuum oven at 37 °C for two hours and stored in a desiccator at room temperature. Thirty microliters each of dDNA and cDNA conjugates were dispensed separately on the NC membrane using a dispensing machine (Biodot XYZ3050) which formed the test and control lines, respectively. These lines were 3 mm apart. The NC membrane was then dried in the vacuum oven at 37 °C for 1 h. The pre-treated sample pad, fiberglass conjugate pad, and cellulose fiber absorbent pad were assembled on the adhesive backing card containing the oven-dried NC membrane. Each component overlapped ~2 mm. The assembled components were cut into 4-mm-wide strips using a programmable shear (Kinematic Automation Matrix 2360), packed in resealable foil pouch with desiccant, and stored at 4 °C until use. Before the assay, desired volume of purified AuNP/rDNA was loaded on the conjugate pad and dried for 1 h at room temperature.

### 2.6. Assay Protocol

Desired volume, × µL of test solution was applied on the sample pad followed by (100-*x*) µL of running buffer containing saline sodium citrate (SSC) and formamide. The appearance of red bands on both test and control lines were evaluated after 20 min: red bands on both test and control lines means a positive (+) result; red band on the control line but nothing on the test line means a negative (-) result; and absence of red band on the control line means test is invalid. 

### 2.7. Quantitation of LFB Signal

Images of the LFB strips after visualization were acquired using scanner/printer (Epson Stylus NX430) and analyzed using ImageJ program developed by the National Institutes of Health (NIH) available online. In ImageJ program, the red signals were converted to 8-bit grey scale and the intensities were converted into profile plots where one peak in the plot represented one red band on the LFB. The intensity of the LFB signal was expressed as peak area.

### 2.8. DENV-1 Strain

Dengue Type 1 virus (Strain: Hawaii, catalog number 0810088CF) in Vero cell line was purchased from ZeptoMetrix Corporation (Buffalo, NY) with analysis certification of 1.7 × 10^5^ U/mL Tissue Culture Infective Dose (TCID_50_). The equivalent plaque-forming value (pfu) was calculated using the formula PFU = 0.7 TCID_50_. 

### 2.9. Extraction and Amplification of DENV-1 RNA

Genomic RNA was extracted from test samples with known pfu/mL of virus using QIAamp^®^ Viral RNA Mini Kit (Qiagen, Louisville, CA) and following manufacturer’s instructions. This extraction procedure utilized 140 µL starting material input and eluted RNA in 60 µL final volume. Extracted viral RNA was amplified using NASBA Liquid Kit Complete from Life Sciences Advanced Technologies, Inc. (St. Petersburg, FL). The amplification was performed in 20 µL reaction mixture volume containing 6.7 µL of buffer (trisHCl, pH 8.5, MgCl_2_, KCl, DTT, dimethyl sulfoxide), 3.3 µL 6× nucleotide mix, 2.0 µL sterile water, 1.0 µL of each 5 µM primers 1& 2, 1.0 µL RNA sample, and 5.0 µL enzyme cocktail (AMV RT, RNase H, T7 RNAP, 0.48 mg/mL BSA in high MW sugar matrix). Extracted viral RNA was amplified using SKU: NWK-1 (NASBA Liquid Kit Complete) from Life Sciences Advanced Technologies, Inc. following manufacturer’s instructions. The amplification was performed in 20 µL reaction mixture volume containing 6.7 µL of buffer (trisHCl, pH 8.5, MgCl_2_, KCl, DTT, dimethyl sulfoxide), 3.3 µL 6X nucleotide mix, 2.0 µL sterile water, 1.0 µL of each 5 µM primers 1& 2, 1.0 µL RNA sample, and 5.0 µL enzyme cocktail (AMV RT, RNase H, T7 RNAP, 0.48 mg/mL BSA in high MW sugar matrix). Briefly, 6.7 µL of the reaction buffer and 3.3 µL nucleotide mixture were mixed well in sterile PCR tube and heated to 41 °C for 5 min. A 4.0 µL of primer mixture containing 2.0 µL water, and 1.0 µL each of 5 µM primers 1& 2 were added. A 1.0 µL of target sample was then added. After gentle mixing in a mini-fuge, annealing was accomplished by heating the RNA-primer-buffer mixture for 2 min at 65 °C and cooling for 10 min at 41 °C. Immediately after annealing, the RNA–primer–buffer mixture was pre-incubated at 41 °C for 1.5 min. The 5.0 µL enzyme cocktail was added and the amplification reaction mixture was incubated at 41 °C for 90 min. The NASBA amplicons was purified using RNeasy^®^ MinElute^®^ Clean-up Kit (Qiagen, Louisville, KY) and following manufacturer’s instructions. A chosen volume of amplification product was adjusted to a volume of 100 µL using RNase-free water, and generated a 12 µL purified RNA eluate. 

## 3. Results and Discussion

### 3.1. Principle of Detection

The principle of detection is based on nucleic acid sandwich reaction on the LFB strip. Before the assay, pad-2 appeared red due to pre-loaded AuNP/rDNA while both test and control lines on pad-3 were invisible. When the sample containing dengue-1 RNA and the running buffer were applied to pad-1, the liquid mixture migrated along the strip from pad-1 through pad-4 by capillary action (Scheme 1B). As the liquid flowed to pad-2, the AuNP/rDNA hybridized with the target RNA and formed the AuNP/rDNA-RNA complex. As the liquid continued to migrate along pad-3, the AuNP/rDNA-RNA hybrid was captured on the test line by a second hybridization reaction between the target RNA and the immobilized dDNA forming the AuNP/rDNA-RNA-dDNA sandwich complex. The sandwich formation accumulated the AuNP label on the test line and was visualized as the first distinct red band on the NC membrane. The excess and unbound AuNP/rDNA from pad-2 continued to migrate to pad-3. These excess probes were captured by a hybridization reaction between rDNA and cDNA forming the AuNP/rDNA-cDNA complex at the control line. This complex formation accumulated the AuNP label at the control line and was visualized as the second distinct red band. In the absence of DENV-1 target, only the red control line was observed. Assay time was 20 min.

Figure 1 shows typical images of valid LFB results with their corresponding profile plots on their right as generated from ImageJ analysis, and the resulting histogram of the test line peak areas. The (−) result for water generated one peak in its profile plot while (+) results for T-1 at different concentrations (0.02–006 µM) generated two peaks in their profile plots. Additionally, as the concentration of T-1 increases, the red test line gets darker, and the resulting peak areas in the profile plot increases. 

### 3.2. Optimization of Experimental Conditions

**Figure 2:**
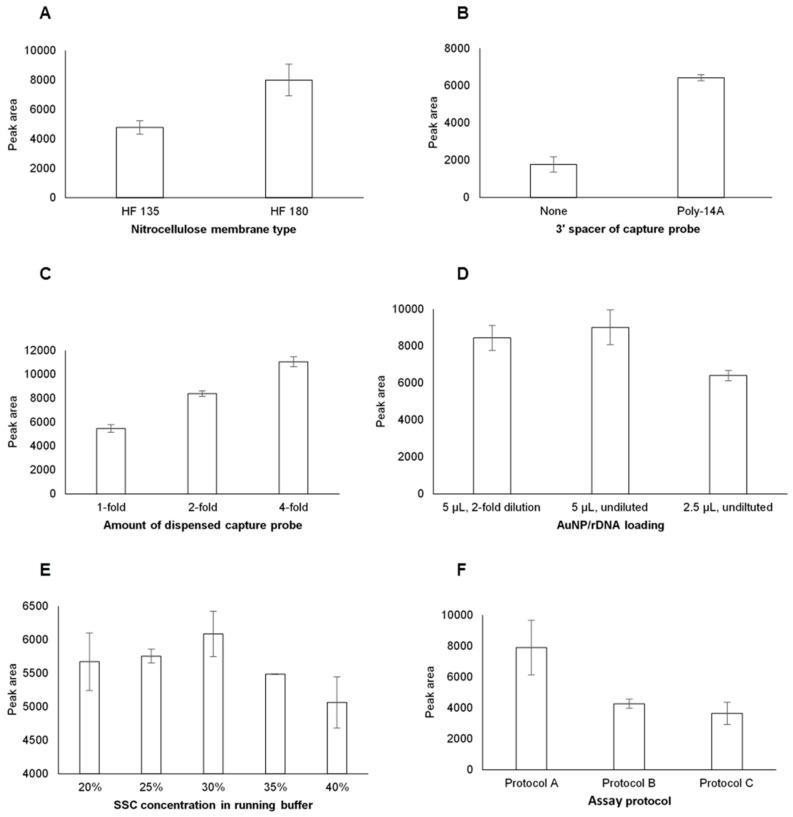
Optimization of LFB parameters. (**A**) Effect of NC membrane type on the signal of LFB; (**B**) Effect of added poly-14A on the signal of LFB; (**C**) Effect of dDNA concentration on the signal of LFB; (**D**) Effect of AuNP/rDNA loading on the signal of LFB; (**E**) Effect of SSC buffer concentration on the signal of LFB; (**F**) Effect of assay protocol on the signal of LFB.

Experimental conditions including type of NC membrane, addition of poly-14A spacer, amount of dDNA conjugate dispensed on NC membrane, AuNP/rDNA conjugate loading on the conjugate pad, and concentrations of SSC and formamide in the running buffer were optimized using 10 µL of 0.05 µM T-1 as model dengue-1 target analyte. Optimal conditions were determined through iterative univariate experiments [22] and evaluating the responses quantitatively. The experimental trial that produced the highest signal expressed in peak area was considered the optimal condition. Membrane type plays a role in the sensitivity of lateral flow biosensors since hybridization reactions are affected by the migration of sample and buffer on the NC membrane [23]. NC membrane types HF 135 and HF 180 with corresponding capillary flow times of 135 and 180 s per 4 cm, respectively, were tested. Figure 2A shows that LFBs with HF 180 NC membrane produced higher signals than those with HF135. The higher signal in HF 180 indicates that longer migration time is favorable. This result is consistent with previous report that NC membrane HFB 24004 with longer migration time produced higher signal compared to HFB 18004 with shorter migration time [21]. 

The addition of poly-14A spacer to the 3′ end of capture probe sequence greatly increased the signal intensity of the LFB. Figure 2B shows a 72% increase in signal intensity using capture probe of similar sequence with poly-14A spacer. The higher signal is attributed to the increased accessibility of the dDNA bases for hybridization as the poly-14A spacer provides dDNA sequence some distance from the biotin–streptavidin anchor. Accessibility of probe sequence was reported as one of the two key factors that affect hybridization in surface-based biosensor [24]. 

The optimal amount of dDNA dispensed on the test line was determined by dispensing 1-, 2-, and 4-fold of the dDNA. Although the 4-fold dDNA gave the highest signal (Figure 2C), the 2-fold dDNA was considered appropriate based on economic advantages. The detection limits of LFBs with 2-fold and 4-fold dDNA were found to be the same at 0.01 µM T-1. Using a 2-fold dDNA would translate to reduced consumption of reagent for the same sensitivity. One of the preferable characteristics for LFB is low cost [25]. Moreover, using 4-fold dDNA translates to the dispensing of conjugate solution 4 times on the NC membrane which causes unwanted overspreading of the solution on the test line. The 2-fold dDNA on the test line was therefore considered optimal. 

The effect of the volume of AuNP/rDNA conjugate loaded to pad-2 was evaluated in preliminary experiments. It was found that the LFB signal increased as the volume of AuNP/rDNA increased. However, this increase was accompanied by increase in noise background. It was observed that loading either 2.5 or 5 µL AuNP/rDNA is a practical possibility considering the following factors: firstly, with a 4-mm wide LFB strip, loading 5 µL or less avoids unwanted overflowing of AuNP/rDNA to adjacent pads. Secondly, loading >5 µL required repeated rounds of dispensing and drying which are considered process downsides. Thirdly, experiments showed that increasing the volume of AuNP/rDNA to higher than 5 µL does not improve the sensitivity of the LFB. In finding the optimal AuNP/rDNA loading, LFBs were tested with varied volumes and dilutions of AuNP/rDNA. Figure 2D demonstrates that for the same 5 µL volume, the LFBs with undiluted AuNP/rDNA gives higher signal than those with 2-fold dilution. Figure 2D also illustrates that for the similarly undiluted AuNP/rDNA, the 5 µL volume produces higher signal. Therefore, 5 µL of undiluted AuNP/rDNA was considered optimal.

Running buffer affects hybridization efficiency of DNA and RNA, minimizes nonspecific adsorption, improves sensitivity, and increases reproducibility [19]. LFBs were tested with varied concentrations of SSC in the running buffer. Figure 2E shows that as SSC concentration increases from 20% to 30%, the signal increases, then decreases as SSC exceeds 30%. Since leaching of the NC membrane was visually observed on assays with 30% SSC, the 25% SSC, which gave the second highest signal, was considered as the optimal concentration. Formamide, a denaturing agent, was added to the running buffer at constant concentration of 4%. The effect of formamide concentrations (2, 4, 8, and 10%) on the signal intensities was determined in a separate experiment. Results demonstrated highest signal at 4% formamide, although t-test indicated that the signals are insignificantly different. For the rest of the tests, assays were done using a running buffer containing 25% SSC and 4% formamide. Hybridization reaction is the most vital process in DNA and RNA biosensors and is affected by ionic strength, sequence composition, and temperature [26]. Formamide is known to decrease annealing temperature between probes and target sequences, and has been used in in situ hybridization to reduce non-specific binding [27,28]. This decrease in annealing temperature is attributed to the ability of formamide to compete with the hydrogen bonds between Watson–Crick base pairs and suppresses secondary structure. A study on the influence of formamide on microarray surface hybridization reported an average decrease of 0.58 ± 0.05 °C/% *v*/*v* and improved hybridization rates which were independent of probe sequence [29]. 

The effect of assay protocol was determined by introducing 10 µL of T-1 and 90 µL running buffer in three different ways. In protocol A, T-1 was first applied to the sample pad and followed by addition of 90 µL running buffer. In protocol B, T-1 was diluted by adding 40 µL of running buffer and then applied to the sample pad followed by addition of 50 µL running buffer. In protocol C, T-1 was diluted by adding 90 µL of running buffer and then applied to the sample pad. Figure 2F demonstrates that direct application of 10 µL test sample followed by 90 µL running buffer gives the highest signal. Results showed that the differences in the signal intensities of protocols B and C were statistically insignificant (*p =* 0.67, *n* = 3), were both lower than and significantly different from protocol A (*p* = 0.03, *n* = 3). In this study, protocol A was used in all assays for detection of dengue-1 RNA.

### 3.3. Analytical Characteristics

**Figure 3:**
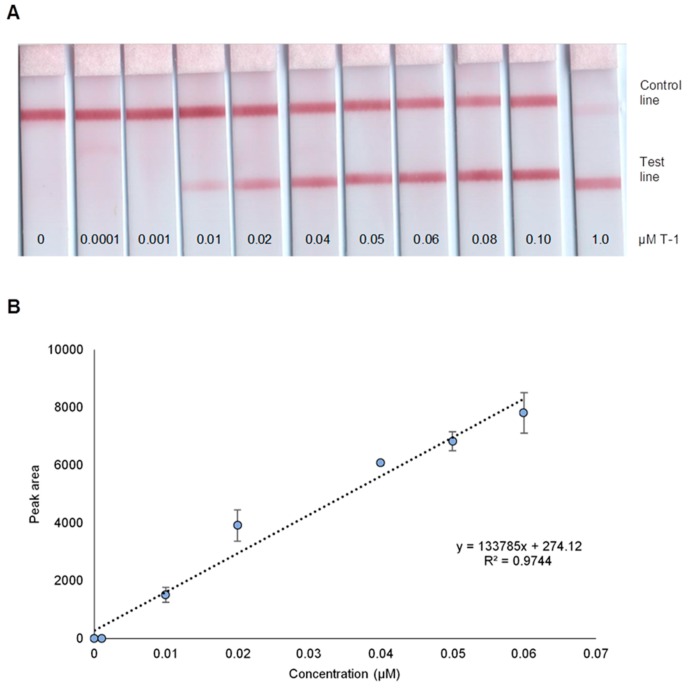
(**A**) Photo images of LFB at varied T-1 concentrations as labeled. (**B**) Calibration curve under optimal conditions.

The analytical performance of the LFB was evaluated at varied concentrations of T-1 under optimal conditions. Figure 3A presents photo images of LFB from 0 to 1.0 µM T-1 with the red test line visible in as low as 0.01µM. The biosensor being developed is intended to generate either “yes” or “no” response in terms of the presence of dengue-1 target in the sample. The cut-off value of a biosensor with visual detection is defined as the lowest concentration where a red band is visible in the test line [30]. Based on this definition, the cut-off value for the LFB is 0.01 µM. The calibration curve (Figure 3B) obtained by plotting the test line peak area versus T-1 concentration shows that the LFB has a linear dynamic range of 0.01–0.06 µM. 

### 3.4. Selectivity Tests

**Figure 4:**
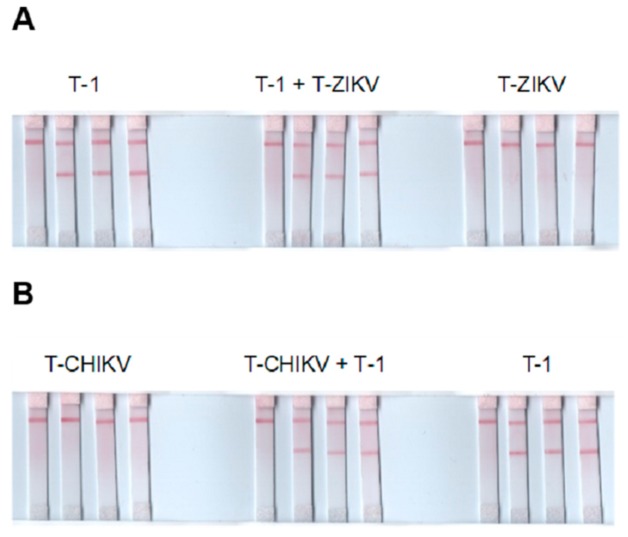
Selectivity test results. Sets of LFB tested with water and triplicates of 0.05 µM target as labeled (**A**) and (**B**).

Selectivity tests were performed in two set-ups. The first was performed against chikungunya and zika viruses using synthetic targets T-CHIKV and T-ZIKV, respectively. These viruses were chosen because they too are transmitted by *Aedes* mosquitoes species and also pathogenic [31,32,33,34]. Solutions of T-1, T-ZIKV, T-CHIKV, mixture of T-1 and T-ZIKV, and mixture of T-1 and T-CHIKV, each containing the same concentration of the targets were tested on LFB. Figure 4A shows (+) result for T-1, (+) result for mixture of T-1 and T-ZIKV, but (−) result for T-ZIKV. These results demonstrate that the LFB is able to discriminate T-1 without interferences from T-ZIKV. Similarly, Figure 4B demonstrates that the LFB was also able to discriminate T-1 without interferences from T-CHIKV. 

**Figure 5:**
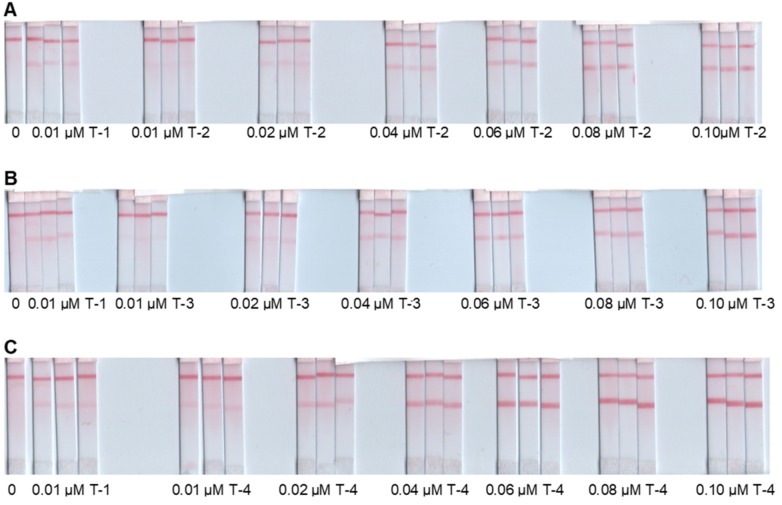
Results of selectivity tests against dengue serotypes 2, 3, and 4. Sets of LFB responses for blank (0), triplicates of 0.01 µM T-1, and triplicates of the different concentrations of (**A**) T-2, (**B**) T-3, and (**C**) T-4 as labeled.

The second selectivity test was performed against dengue serotypes 2, 3, and 4 using T-2, T-3, and T-4, respectively. Cross-reactivity was evaluated at varied concentrations of T-2, T-3, and T-4 (0.01, 0.02, 0.04, 0.06, 0.08, and 0.1 µM). Figure 5 presents three sets (A, B, and C) of LFB responses, each set containing a blank, triplicates of 0.01 µM T-1, and triplicates of the different concentrations of other dengue serotype. Figure 5A,B demonstrate cross reactions with T-2 and T-3, respectively, starting at 0.02 µM of the serotype target. These cross reactions are both above the LFB cut-off value of 0.01 µM T-1. Figure 5C showed cross reaction with T-4 starting at 0.01 µM.

### 3.5. Detection of Dengue-1 Viral RNA 

Proof-of-concept application was done in two set-ups. In the first set-up, dengue-1 virus culture fluid was serially diluted with water and obtained 1.2 × 10^4^, 1.2 × 10^3^, and 1.2 × 10^2^ pfu/mL viral concentrations. After RNA extraction, NASBA, and purification, 5 µL amplicon was tested on the LFB along with negative controls, T-RNA, and T-1 at varied concentrations. Results are summarized in Table 2.

The LFB produced (−) results for all negative controls, 1.2 × 10^2^ and 1.2 × 10^3^ pfu/mL, but produced (+) results for 1.2 × 10^4^ pfu/mL and synthetic targets. From Table 2, the LFB cut-off value was determined to be at 1.2 × 10 ^4^ pfu/mL. In another detection experiment, similar viral RNA extraction, NASBA, and purification were performed for 1.2 × 10^4^ pfu/mL and 4.3 × 10^4^ pfu/mL. Three sets of water were used as negative controls: one for extraction, another for NASBA, and another for LFB assay. T-1 at varied concentrations were simultaneously ran during LFB assays. Results in Figure S1 showed that LFB produced (−) results for all three negative controls, and (+) results for all positive controls. Quantitatively, the higher peak area for 4.3 × 10^4^ pfu/mL compared to 1.2 × 10^4^ pfu/mL demonstrates the sensitivity of the LFB to the changes in virus concentration as it does to the changes in concentration of synthetic targets.

**Figure 6:**
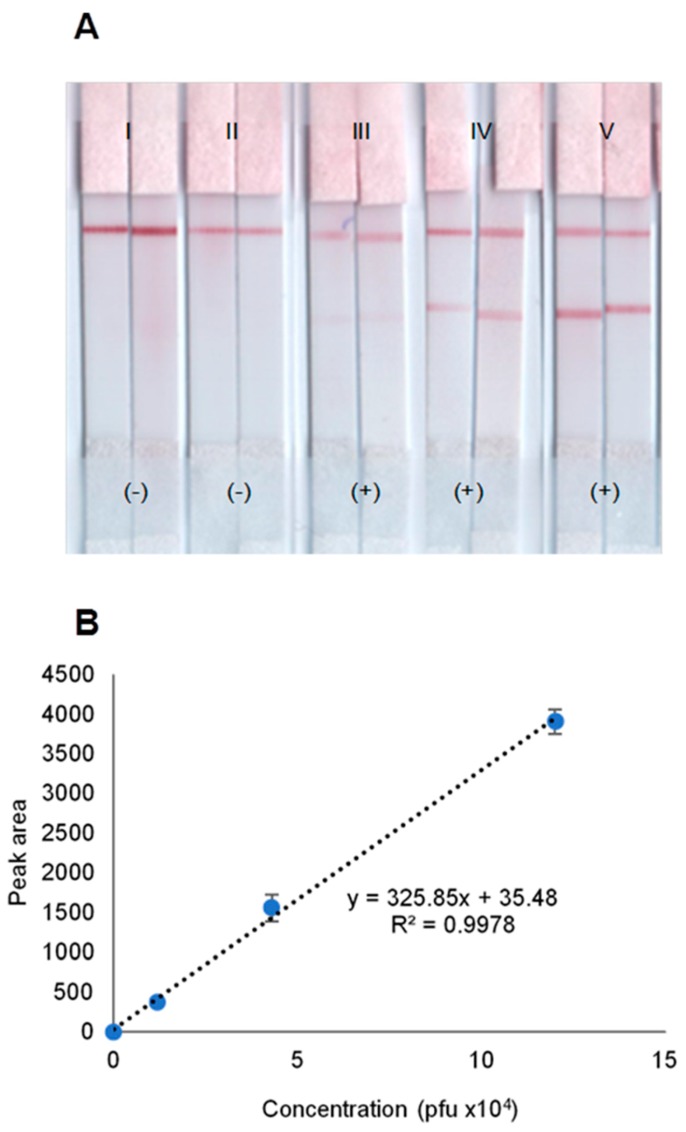
(**A**) Photo images of replicate LFB strips tested with NASBA products showing (−) results for water (I) and pooled human sera (II), but (+) results for DENV-1 in pooled human sera at 1.2 × 10^4^ (III), 4.3 × 10^4^ (IV) and 1.2 × 10^5^ pfu /mL (V), and (**B**) their corresponding dose–response curve.

Finally, the LFB was challenged by detecting dengue-1 virus infection in pooled human sera at different viral loads. Dengue-1 virus culture fluid was diluted with pooled human sera and obtained 0, 1.2 × 10^4^, 4.3 × 10^4^, and 1.2 × 10^5^ pfu/mL viral concentrations. After RNA extraction and amplification, purified 5 µL amplicons were diluted to 10 µL and tested on the LFB. Figure 6A demonstrates that the LFB gives (−) results for all negative controls (water and human sera) and gives (+) results for all positive controls. The LFB responses showed one red line for the absence and two lines for the presence of DENV-1 in pooled human sera. Notably, the red test line intensity increases as the virus concentration increases. The corresponding dose–response curve in Figure 6B shows the coefficient of determination value of 0.9978 (*n* = 2).

In summary, the LFB coupled with RNA extraction and amplification was able to detect the presence of dengue-1 virus in pooled human sera with a cut-off value of 1.2 × 10^4^ pfu/mL. The observed cross reactivity with other dengue serotypes might be considered as downside for wanting to specifically detect dengue-1, but it could be a promising biosensor for generalized dengue infection diagnostic screening. A work using NASBA assay with silica RNA extraction and electrochemiluminescence detection reported that detection limits for the four dengue serotypes ranged from 1 to 10 pfu/mL [18], which are very low compared to the cut-off value of 1.2 × 10^4^ pfu/mL in the present study. Improvement in this aspect can be explored in the future by AuNP signal amplification using either enzyme or silver. Improved sensitivity of lateral flow strip was shown using horseradish peroxidase enzyme as AuNP co-label [23]. Deposition of silver on AuNP after the usual lateral flow color development improved the limit of detection [35,36].

## 4. Conclusions

A colorimetric lateral flow biosensor was developed for the visual detection of DENV-1 RNA. The study demonstrates for the first time the applicability of dextrin-capped AuNP as label in lateral flow assay. The presence of DENV-1 target RNA was easily detected by observing the appearance of a red band on the test line of the LFB strip. The use of NASBA generates single stranded negative-sense RNA amplicons without the need for costly PCR thermocycling equipment and can be directly applied to the LFB for detection. Proof-of-concept application demonstrated the capability of the biosensor to detect DENV-1 infection in pooled human sera with a cut-off value of 1.2 × 10^4^ pfu/mL. Coupled with viral RNA extraction and NASBA, target dengue-1 RNA was detected on the LFB in 20 min. The development of this LFB provides a promising diagnostic platform for the simple detection of dengue infection in high-risk, resource-limited areas.

## Figures and Tables

**Table 1:** DNA sequences of probes and primers used in this study.

Oligonucleotide	Sequence
DENV-1 probe	5′-GGGAAGCTGTATCCTGGTGGTAAGG(A)_14_-3′-biotin
Reporter probe	Thiol-5′-GATGCAAGGTCGCATATGAG-3′
Control probe	Biotin-5′-CTCATATGCGACCTTGCATC-3′
Primer 1 ^[a]^	5′-aattctaatacgactcactatagggGAGACAGCAGGATCTCTGGTCT-3′
Primer 2 ^[b]^	5′-gatgcaaggtcgcatatgagGGTTAGAGGAGACCCCTCCC-3′

^[a]^ Lowercase bases represent T7 promoter sequence. ^[b]^ Lowercase bases are homologous to reporter probe.

**Table 2:** Summary of detection of DENV-1 in aqueous solution.

Sample	Concentration	LFB result (+/−)
(−) LFB control	0	(−)
(−) Extraction control	0	(−)
(−) NASBA control	0	(−)
DENV-1	1.2 × 10^2^ pfu/mL	(−)
DENV-1	1.2 × 10^3^ pfu/mL	(−)
DENV-1	1.2 × 10^4^ pfu/mL	(+)
T-RNA	0.1 µM	(+)
T-1	0.02–0.08 µM	All (+)

## Supplementary Materials

It can be found at https://www.mdpi.com/2075-4418/9/3/74/s1.
