# Supplementary material for: Visual Detection of Dengue-1 RNA Using Gold Nanoparticle-Based Lateral Flow Biosensor
Source: Diagnostics (Basel). 2019 Jul 11;9(3):74. doi: 10.3390/diagnostics9030074 (PMC6787709; doi:10.3390/diagnostics9030074)
Supplement: Supplementary file 1 [file diagnostics-09-00074-s001.pdf]

Supplementary Material

A

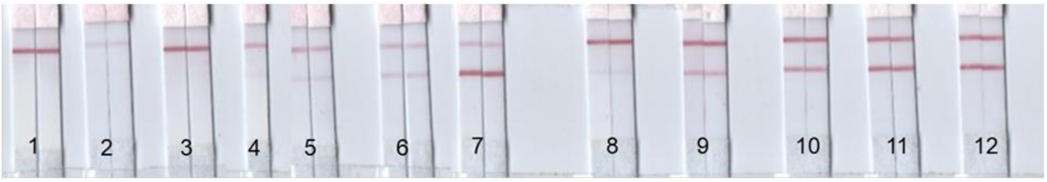

B

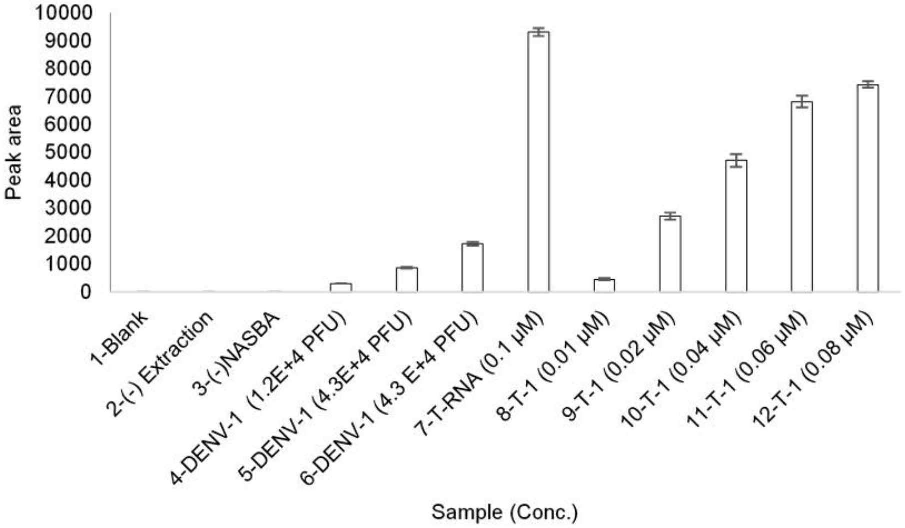

Figure 1. (**A**) Photo images of LFB strips tested with different samples and (**B**) the corresponding histogram. The number sequence of LFB strips corresponds to the same sequence in the histogram.
